# Childhood maltreatment and appearance-related social media consciousness: the moderating role of gender and social media use time

**DOI:** 10.3389/fpsyg.2026.1865576

**Published:** 2026-09-29

**Authors:** Weiying Gao, Wanran Huang

**Affiliations:** 1 School of Journalism and Communication, South China University of Technology, Guangzhou, China; 2 Department of Philosophy, Xiamen University, Xiamen, China

**Keywords:** appearance-related social media consciousness, body image, childhood maltreatment, gender, social media use time

## Abstract

**Objective:**

Appearance-related social media consciousness (ASMC) has emerged as an important construct for understanding appearance-related vulnerability in social media contexts, yet its developmental antecedents remain underexplored. This study examined the associations between retrospectively reported childhood maltreatment and ASMC and tested whether these associations varied by gender and social media use time.

**Methods:**

Participants were 405 Chinese university students recruited from two universities. They completed measures of ASMC, childhood maltreatment, social media use time, and demographic characteristics. Path analysis, multi-group analysis, and moderation analysis were conducted, with body mass index (BMI) controlled.

**Results:**

Emotional abuse (*β* = 0.12, *p* = 0.040) and sexual abuse (*β* = 0.12, *p* = 0.014) were positively associated with ASMC, whereas physical neglect was negatively associated with ASMC (*β* = −0.13, *p* = 0.047). The overall pattern of maltreatment–ASMC associations differed by gender, Δ*χ*^2^(5) = 11.50, *p* = 0.042. Direct Wald tests indicated that only the sexual abuse–ASMC association differed significantly between males and females, *χ*^2^(1) = 9.61, *p* = 0.002.

**Conclusion:**

These findings suggest that retrospectively reported childhood maltreatment is associated with ASMC, with different maltreatment subtypes showing distinct patterns of association. The sexual abuse–ASMC association differed significantly by gender, whereas no significant moderating effect of social media use time was found.

## 1 Introduction

With the widespread use of image and short video–based platforms, social media has become an important space for college students to engage in self-presentation, social interaction, and peer evaluation (Pang, 2020; Yang and Li, 2014). Unlike earlier online environments that were primarily text-based, contemporary social media heavily relies on photos, short videos, filters, beautification features, and interactive feedback (Eshiet, 2020; Siddiqui, 2021), making physical appearance an increasingly salient cue in online communication. Previous research has shown that appearance-focused social media environments not only strengthen individuals’ attention to their appearance but may also increase body image concerns and risks to psychological adjustment (Cohen et al., 2017; Kagan et al., 2025; Watson, 2022).

Against this background, appearance-related social media consciousness (ASMC) has gradually emerged as an important concept for understanding appearance-related risks on social media. ASMC emphasizes individuals’ ongoing concern with whether they “look attractive” in social media contexts, as well as their cognitive, emotional, and behavioral experiences regarding how others may evaluate their online appearance (Choukas-Bradley et al., 2020). It is reflected not only in repeated inspection, editing, and management of one’s appearance in photos, but also in expectations and concerns about audience feedback. Existing research has shown that ASMC is significantly associated with body surveillance, depressive symptoms, disordered eating, and psychological distress, suggesting that it is not merely general appearance dissatisfaction, but a risk-related psychological experience closely tied to social media interaction processes (Choukas-Bradley et al., 2020; Maheux et al., 2024; Tie et al., 2026).

### 1.1 Childhood maltreatment and appearance-related social media consciousness

It is worth noting that an increasing number of studies suggest that relying solely on social media use time to explain psychological outcomes is insufficient (Maheux et al., 2025; Tie et al., 2026). Social media is not a unitary exposure; different types of content, interaction patterns, and psychological processing may be associated with distinct risk profiles (Maheux et al., 2025; Oprea and Bâra, 2025). Compared with the broad indicator of screen time, ASMC is better able to capture individuals’ specific appearance-related cognitive and emotional experiences on social media, and thus is more helpful in explaining the links between social media use, body image, and mental health (Maheux et al., 2024; Tie et al., 2026). However, compared with research on the consequences of ASMC and its measurement, research on its antecedents remains clearly limited. Existing studies have only begun to explore the formation of online appearance preoccupation from perspectives such as appearance teasing, appearance-based rejection sensitivity, and coping strategies; overall, this line of research is still at an early stage.

From a developmental perspective, childhood maltreatment may be an important antecedent for understanding the formation of ASMC. Childhood maltreatment is not a single experience, but a multidimensional construct comprising several subtypes, typically including emotional abuse, physical abuse, sexual abuse, emotional neglect, and physical neglect (Bernstein et al., 2003). Existing research has shown that different types of childhood maltreatment do not have identical effects on subsequent psychological and behavioral problems; combining them into a single total score may obscure the unique effects of specific subtypes (Burns et al., 2012; Dawson et al., 2022; Monteleone et al., 2019; Tie et al., 2025). Prior research has also adopted this approach, emphasizing that different subtypes of childhood maltreatment may be associated with distinct later psychological and behavioral outcomes (Cicchetti and Handley, 2019), therefore should be examined separately.

One possible theoretical interpretation of the association between childhood maltreatment and ASMC can be drawn from schema theory. Schema theory proposes that individuals gradually develop deep cognitive structures about the self, others, and relationships through early interactions with significant others; when children are repeatedly exposed to experiences such as devaluation, humiliation, neglect, or harm, they are more likely to develop early maladaptive schemas, such as defectiveness/shame, social isolation, and unlovability (Shorey et al., 2013). A systematic review further indicates that childhood emotional abuse and emotional neglect are relatively consistently associated with maladaptive schemas, and that these associations emerge before adulthood (May et al., 2022). When individuals chronically understand themselves as “not good enough” or “easily rejected,” they are more likely, in highly visible, comparable, and evaluative environments such as social media, to treat appearance as an important cue of self-worth. Because the core of ASMC is not only “how I view my own appearance,” but more importantly “how others will evaluate my appearance” (Choukas-Bradley et al., 2020). Individuals with stronger negative self-schemas and shame tendencies may more likely to repeatedly worry, when taking photos, posting images, and browsing content, about whether they are attractive enough and whether they will receive negative evaluations, thereby exhibiting higher levels of ASMC. From this theoretical perspective, subtypes such as emotional abuse and emotional neglect, which more directly undermine self-worth, may be particularly important to consider.

Objectification theory provides another possible framework for interpreting the association between childhood maltreatment and ASMC. Objectification theory suggests that in social environments where individuals are continuously observed and evaluated, they may gradually adopt an internalized observer’s perspective toward their own bodies, thereby developing self-objectification and body surveillance (Fredrickson and Roberts, 1997). This theory has since been widely used to explain the relationships among appearance-related media, body surveillance, body shame, and body dissatisfaction. A recent meta-analysis shows that self-objectification is moderately and positively associated with body dissatisfaction and body shame (Saunders et al., 2024). At the same time, systematic reviews and meta-analyses have indicated that childhood maltreatment is consistently associated with cognitive–affective body image difficulties in adulthood, suggesting that early adversity can profoundly shape individuals’ feelings, evaluations, and experiences related to their bodies (Bödicker et al., 2022). Based on this, it can be inferred that childhood maltreatment may make individuals more likely to develop an externalized perspective on their bodies and appearance, while the highly visual nature of social media may further reinforce this tendency of “seeing oneself from the outside,” extending body surveillance into a continuous concern with and management of one’s online appearance—that is, higher levels of ASMC. Moreover, different types of childhood maltreatment may vary in the extent to which they contribute to body surveillance and appearance-focused self-attention.

Taken together, schema theory and objectification theory provide two possible frameworks for understanding why childhood maltreatment may be associated with ASMC. Negative self-schemas, shame, self-objectification, and body surveillance may represent processes worth examining in future research; however, these variables were not measured in the present study. Accordingly, the present study focuses on subtype-specific associations rather than testing the mechanisms underlying these associations.

### 1.2 The moderating role of gender

In addition, the association between childhood maltreatment and ASMC may vary according to individual characteristics and the level of media exposure. First, gender may be an important moderating factor. Existing research on ASMC generally finds that females report higher average levels of ASMC than males and tend to show greater vulnerability to appearance-related pressures and body image concerns (Choukas-Bradley et al., 2020; Tie et al., 2024). At the same time, however, longitudinal studies have not consistently found that gender significantly alters the strength of the association between ASMC and adjustment outcomes (Maheux et al., 2024; Tie et al., 2026). Therefore, whether gender amplifies the risk linking childhood maltreatment to ASMC, or mainly reflects mean-level differences, remains to be further examined.

### 1.3 The moderating role of social media use time

Social media use time may also shape the association between childhood maltreatment and ASMC. Although prior research has emphasized that social media effects cannot be explained solely by usage duration, this does not imply that time spent is unimportant (Kross et al., 2021; Orben and Przybylski, 2019). Longer social media use generally indicates greater exposure to appearance-focused content, more opportunities for social comparison, and greater involvement in appearance-centered online interactions (Cohen and Blaszczynski, 2015; Fardouly and Vartanian, 2016). For individuals who are already more sensitive to external evaluation due to childhood maltreatment, prolonged immersion in social media environments may further intensify appearance monitoring and appearance-related concerns (Fardouly and Vartanian, 2016; May et al., 2022). As a result, social media use time may strengthen the association between childhood maltreatment and ASMC. At the same time, ASMC reflects a more specific appearance-related risk experience than general usage indicators. Thus, compared with overall time spent on social media, ASMC may better capture the psychological processes through which social media becomes linked to appearance-related vulnerability.

### 1.4 The present study

Taken together, existing research has shown that ASMC is an important construct for understanding the links between social media use, body image, and psychological adjustment. However, the current literature has mainly focused on its measurement and outcome variables, with relatively limited attention to its antecedent mechanisms. In particular, childhood maltreatment, as an important form of early adversity, may increase individuals’ appearance-related consciousness on social media through two theoretical pathways: negative self-schemas and objectification–body surveillance. Different types of childhood maltreatment may also show distinct patterns of association. However, direct evidence for these mechanisms remains limited. At the same time, gender and social media use time may further shape this association. Based on this, the present study aims to examine the relationships between overall childhood maltreatment, its subtypes, and ASMC. It also tests the moderating roles of gender and social media use time. The goal is to provide a more nuanced developmental perspective on the formation of ASMC (Figure 1). The following hypotheses are proposed:

**Figure 1:**
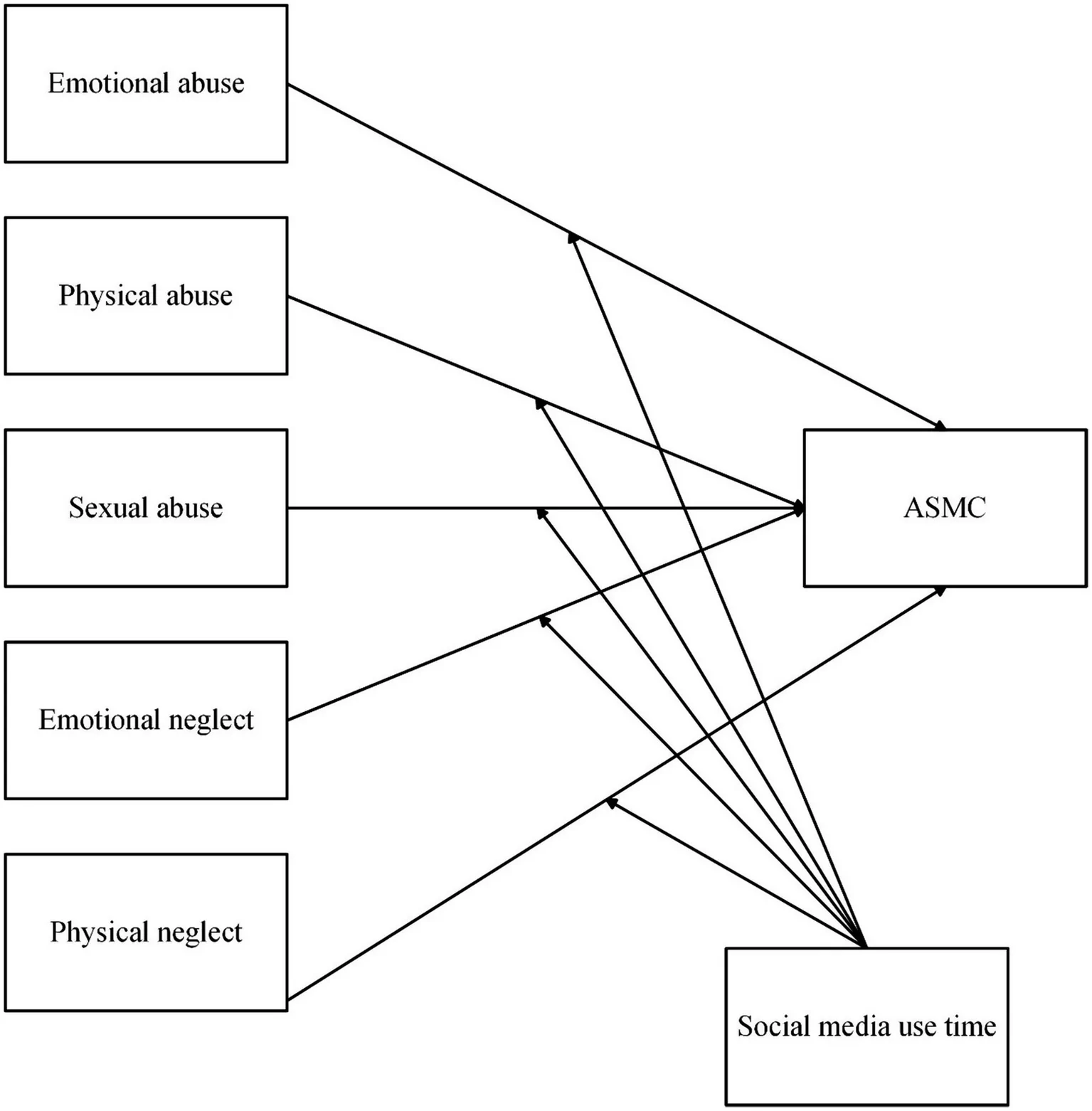
Conceptual model.

*H1*: Different types of childhood maltreatment are differentially associated with ASMC.

*H2*: Gender moderates the association between childhood maltreatment and ASMC.

*H3*: Social media use time moderates the association between childhood maltreatment and ASMC.

## 2 Methods

### 2.1 Participants and procedure

This study was approved by the Ethics Committee of Xiamen University and complied with the principles of the Declaration of Helsinki (1964) and its subsequent revisions. Participants were students recruited from two universities in Wuhan and Nanjing, China with 210 participants recruited from one university and 240 from the other. Data collection was conducted in September 2025. Eligible participants were current university students who provided informed consent to participate. Before completing the survey, all participants were presented with an online informed consent form outlining the study purpose, the voluntary and anonymous nature of participation, and the intended use of the data. Participants were informed that they could discontinue the survey at any time without penalty. Participants were informed that they would receive RMB 5 upon completion of the survey. Only those who provided consent were allowed to proceed.

Data were collected through the Wenjuanxing platform (www.wjx.cn). The questionnaire was designed such that all items required a response before submission, thereby preventing missing data. In addition, the platform restricted each IP address to a single submission to enhance data quality. The survey link was distributed via university-based social media channels and began with the informed consent page. A total of 450 responses were initially obtained. An instructed-response attention-check item was included in the questionnaire, asking attentive participants to select “strongly agree.” The exclusion criterion was specified prior to the statistical analyses. Participants who failed the attention check were excluded. After excluding 45 participants who failed the attention check, the final sample comprised 405 participants, including 189 from one university and 216 from the other. The final sample included 155 males (38.3%) and 250 females (61.7%). Participants had a mean age of 19.56 years (SD = 1.04, range = 18–21 years).

### 2.2 Measures

#### 2.2.1 Demographics

Demographic characteristics were obtained through self-report. Participants indicated their age in years and reported their gender using two categories (male or female). In addition, they provided their height (cm) and weight (kg). BMI ranged from 13.84 to 28.46 (M = 20.65, SD = 2.69).

#### 2.2.2 Appearance-related social media consciousness

To assess ASMC, we employed the Appearance-Related Social Media Consciousness Scale (Tie et al., 2024). The Chinese version of this scale was adapted from (Tie et al., 2024). The scale consists of 13 items, each rated on a 7-point Likert scale ranging from 1 (Never) to 7 (Always). Mean scores across items were computed, with higher scores representing stronger levels of ASMC. Previous research has demonstrated that the Chinese version of the ASMC exhibits good psychometric properties in Chinese university student sample (Tie et al., 2024). In the present study, the scale showed excellent internal consistency (Cronbach’s *α* = 0.92).

#### 2.2.3 Childhood maltreatment

To assess childhood maltreatment experiences, we used the short form of the Childhood Trauma Questionnaire (CTQ-SF), a widely used self-report measure of early adverse experiences (Bernstein et al., 2003). The CTQ-SF includes 28 items, among which three items are designed to evaluate response validity. All items are rated on a 5-point Likert scale ranging from 1 (Never) to 5 (Always). The scale comprises five dimensions: emotional abuse, physical abuse, sexual abuse, emotional neglect, and physical neglect. Subscale scores are obtained by summing the corresponding item responses, with higher scores indicating more severe maltreatment experiences. Previous studies have demonstrated that the Chinese version of the CTQ-SF has sound psychometric properties in Chinese young adult samples (He et al., 2019). In the present study, the overall internal consistency of the scale was acceptable (Cronbach’s *α* = 0.80). In the present study, internal consistency was assessed separately for each subscale using Cronbach’s α and McDonald’s ω. The reliability coefficients were 0.71 and 0.71 for physical neglect, 0.72 and 0.69 for emotional abuse, 0.75 and 0.79 for emotional neglect, 0.77 and 0.70 for physical abuse, and 0.73 and 0.77 for sexual abuse, respectively. The three minimization/denial items were not included in the substantive analyses and were excluded from the calculation of the five maltreatment subscale scores.

#### 2.2.4 Social media use time

Social media use time was assessed with a single item: “Overall, how long do you spend on social media platforms on a typical day?” Responses were coded on a 7-point ordinal scale: 1 = 5 min or less, 2 = 15 min, 3 = 30 min, 4 = 1 h, 5 = 2 h, 6 = 4 h, and 7 = 6 h or more. Higher scores reflected greater levels of social media use time. Similar approaches using single-item, category-based or Likert-type measures to assess time spent on social media have been widely adopted in prior research (Ni et al., 2020; Salerno et al., 2025; Sametoglu, 2025). In the present study, social media use scores were moderately distributed (*M* = 3.96, SD = 1.25, median = 4, IQR = 2, skewness = 0.61).

### 2.3 Data analysis

All statistical analyses were conducted using R (version 4.1.2). Descriptive statistics (means and standard deviations) and Pearson correlation coefficients were calculated for all study variables, including childhood maltreatment dimensions (physical neglect, emotional abuse, emotional neglect, physical abuse, and sexual abuse), social media use time, and ASMC. The primary model simultaneously included all five childhood maltreatment subtypes as predictors of ASMC, with BMI as a covariate. Gender and social media use time were examined as focal moderators rather than routine covariates, whereas age and university/site were not included because of the restricted age range and their non-focal role in the study. The model was estimated using lavaan with robust maximum likelihood estimation (MLR). Multicollinearity was assessed using variance inflation factors (VIFs) and tolerance values.

Before conducting gender-based structural comparisons, measurement invariance of the ASMC across gender was examined sequentially at the configural, metric, and scalar levels. Changes in CFI, RMSEA, and SRMR were considered in evaluating measurement invariance. All five dimensions of childhood maltreatment were simultaneously entered as predictors of ASMC. To examine whether the structural relationships differed by gender, multiple-group path analyses were conducted. Specifically, an unconstrained model (in which all structural paths were freely estimated across groups) was compared with a constrained model (in which corresponding paths were set equal across males and females). BMI was freely estimated across groups. Differences between models were evaluated using the scaled chi-square difference test under MLR estimation. A significant Δ*χ*^2^ suggests the presence of gender differences (Cheung and Rensvold, 2002).

All five-childhood maltreatment × social media use time interaction terms were simultaneously included in the same regression model, consistent with Hypothesis 3. ASMC was specified as the dependent variable, and the five childhood maltreatment subtypes, social media use time, and their corresponding interaction terms were entered as predictors. All continuous predictors were standardized prior to constructing the interaction terms. To account for multiple testing across the five interaction effects, Holm-adjusted *p* values were calculated. Body mass index (BMI) was included as a covariate in all analyses.

Sensitivity analyses were additionally conducted to evaluate the robustness of the subtype-specific associations. Each childhood maltreatment subtype was first examined separately with BMI included as a covariate, and these estimates were then compared with those from the mutually adjusted model including all five maltreatment subtypes simultaneously.

## 3 Results

### 3.1 Descriptive analyses

Descriptive statistics and correlations by gender are presented in Table 1. The five dimensions of childhood maltreatment were positively intercorrelated in both groups, with effect sizes ranging from small to moderate. Regarding associations with ASMC, sexual abuse was significantly positively correlated with ASMC among females (*r* = 0.19, *p* = 0.003), but not among males (*r* = −0.001, *p* = 0.988). Other forms of maltreatment were not consistently associated with ASMC across gender groups. Social media use time was positively correlated with ASMC in both males (*r* = 0.19, *p* = 0.016) and females (*r* = 0.24, *p* < 0.001).

**Table 1:** Means, standard deviations, and pearson correlation for the study variables.

Variable	1	2	3	4	5	6	7
1. Physical neglect	—	0.11	0.40***	−0.02	−0.03	−0.003	0.06
2. Emotional abuse	0.32***	—	0.37***	0.46***	0.08	0.03	0.09
3. Emotional neglect	0.64***	0.48***	—	0.27**	0.09	−0.002	0.15
4. Physical abuse	0.20**	0.52***	0.32***	—	−0.04	−0.05	0.003
5. Sexual abuse	0.16**	0.11	0.19**	0.24***	—	−0.05	−0.001
6. Social media use time	−0.01	0.02	0.17**	0.08	0.06	—	0.19*
7. ASMC	−0.08	0.09	0.08	0.05	0.19**	0.24***	—
8. Male M (SD)	6.07 (1.64)	6.16 (2.01)	7.11 (2.31)	5.67 (1.54)	5.16 (0.74)	3.81 (1.16)	2.90 (1.19)
9. Female M (SD)	5.98 (1.80)	6.80 (2.58)	7.49 (2.82)	5.68 (1.83)	5.30 (1.19)	4.05 (1.30)	3.44 (1.04)

Upper triangle = males; lower triangle = females. ASMC = appearance-related social media consciousness. *p < 0.05, **p < 0.01, ***p < 0.001.

### 3.2 Results of path analysis predicting ASMC

Multicollinearity diagnostics indicated no serious concerns, with VIF values ranging from 1.02 to 1.74 and all tolerance values above 0.58. The standardized results of the baseline path model are shown in Table 2. BMI was significantly and negatively associated with ASMC (*β* = −0.14, *p* = 0.004). Regarding childhood maltreatment, physical neglect was negatively associated with ASMC (*β* = −0.13, *p* = 0.047), whereas sexual abuse was positively associated with ASMC (*β* = 0.12, *p* = 0.014). Emotional abuse also showed a significant positive association with ASMC (*β* = 0.12, *p* = 0.040), whereas emotional neglect was positively but not significantly related to ASMC (*β* = 0.10, *p* = 0.073). Physical abuse was not significantly related to ASMC (*β* = −0.03, *p* = 0.612).

**Table 2:** Standardized results of the model predicting ASMC.

Predictor	*β*	SE	95% CI
BMI	−0.14**	0.05	[−0.24, −0.05]
Physical neglect	−0.13*	0.06	[−0.25, −0.002]
Emotional abuse	0.12*	0.06	[0.003, 0.24]
Emotional neglect	0.10	0.06	[−0.01, 0.21]
Physical abuse	−0.03	0.06	[−0.14, 0.08]
Sexual abuse	0.12*	0.05	[0.02, 0.22]

*p < 0.05, **p < 0.01, ***p < 0.001.

Sensitivity analyses were conducted by examining each childhood maltreatment subtype separately with BMI as a covariate. Physical neglect was not significantly associated with ASMC when examined separately (*β* = −0.05, *p* = 0.416), whereas emotional abuse (*β* = 0.12, *p* = 0.006) and sexual abuse (*β* = 0.14, *p* = 0.005) showed positive associations. Emotional neglect showed a marginal association (*β* = 0.09, *p* = 0.050), whereas physical abuse was not significantly associated with ASMC (*β* = 0.05, *p* = 0.230). Notably, the negative association for physical neglect emerged only in the mutually adjusted model including all five maltreatment subtypes (*β* = −0.13, *p* = 0.047), suggesting that this effect should be interpreted as a conditional, model-dependent association.

### 3.3 Gender differences in path analysis predicting ASMC

Before comparing the structural paths across gender, measurement invariance of the ASMC was examined using multi-group CFA with the ASMC items treated as ordered categorical indicators. The results supported measurement invariance across gender. Changes in model fit were small from the configural to the metric model (ΔCFI = −0.003, ΔRMSEA = −0.008, ΔSRMR = 0.003). Constraining item thresholds to equality also produced only small changes in model fit (ΔCFI = 0.010, ΔRMSEA = 0.009, ΔSRMR = −0.003), supporting scalar invariance. Strict invariance was also supported (ΔCFI = −0.002, ΔRMSEA = −0.009, ΔSRMR = 0.003).

To examine whether the path model differed by gender, a multi-group analysis was conducted. A Satorra–Bentler scaled chi-square difference test indicated that constraining the five childhood maltreatment–ASMC paths to equality across gender significantly worsened model fit, Δ*χ*^2^(5) = 11.50, *p* = 0.042, indicating an overall gender difference in the associations between childhood maltreatment subtypes and ASMC.

In the male group, none of the five childhood maltreatment subtypes was significantly associated with ASMC. In the female group, physical neglect was negatively associated with ASMC (*β* = −0.25, *p* = 0.011), whereas emotional neglect (*β* = 0.17, *p* = 0.048) and sexual abuse (*β* = 0.19, *p* < 0.001) were positively associated with ASMC. BMI was not significantly associated with ASMC in either males (*β* = −0.13, *p* = 0.057) or females (*β* = −0.09, *p* = 0.234). To directly examine gender differences, Wald tests were conducted for each maltreatment path. Only the sexual abuse–ASMC association differed significantly between males and females, *χ*^2^(1) = 9.61, *p* = 0.002. The between-group differences were not significant for physical neglect (*p* = 0.081), emotional abuse (*p* = 0.612), emotional neglect (*p* = 0.866), or physical abuse (*p* = 0.752). Thus, although emotional neglect was significant within the female group, it should not be interpreted as a female-specific association; evidence for a gender difference was limited to the sexual abuse–ASMC association (Table 3).

**Table 3:** Multi-group path analysis predicting ASMC by gender.

Predictor	Male *β* [95% CI]	Female *β* [95% CI]	Wald *χ*^2^(1)	*p* for gender difference
Physical neglect	0.01 [−0.17, 0.19]	−0.25 [−0.45, −0.06]	3.04	0.081
Emotional abuse	0.10 [−0.06, 0.27]	0.09 [−0.09, 0.27]	0.26	0.612
Emotional neglect	0.13 [−0.01, 0.27]	0.17 [0.003, 0.35]	0.03	0.866
Physical abuse	−0.06 [−0.25, 0.12]	−0.04 [−0.18, 0.10]	0.1	0.752
Sexual abuse	−0.03 [−0.08, 0.03]	0.19 [0.08, 0.31]	9.61	0.002

*β* = standardized coefficients; ASMC, appearance-related social media consciousness. *p < 0.05, **p < 0.01, ***p < 0.001.

### 3.4 Moderating effect of social media use time

Following the path analysis, moderation analyses were conducted to examine whether social media use time conditioned the associations between the five childhood maltreatment dimensions and ASMC. Consistent with Hypothesis 3, all five childhood maltreatment × social media use time interaction terms were simultaneously entered into the same regression model. To account for multiple testing across the five interaction terms, Holm-adjusted *p* values were calculated.

None of the five interaction effects reached statistical significance after correction for multiple testing. Specifically, the interactions of physical neglect (p_adjusted_ = 0.711), emotional abuse (p_adjusted_ = 1.000), emotional neglect (p_adjusted_ = 0.409), physical abuse (p_adjusted_ = 1.000), and sexual abuse (p_adjusted_ = 0.297) with social media use time were all nonsignificant (Table 4). Thus, the revised analyses did not provide evidence that social media use time moderated the associations between childhood maltreatment subtypes and ASMC, and Hypothesis 3 was not supported (Table 4).

**Table 4:** Moderation analysis predicting ASMC.

Predictor	*β*	SE	*p*	Holm-adjusted *p*	95% CI
BMI	−0.15	0.05	0.002	—	[−0.25, −0.06]
Physical neglect	−0.11	0.06	0.064	—	[−0.23, 0.01]
Emotional abuse	0.13	0.06	0.035	—	[0.01, 0.24]
Emotional neglect	0.09	0.07	0.173	—	[−0.04, 0.22]
Physical abuse	−0.04	0.06	0.521	—	[−0.16, 0.08]
Sexual abuse	0.12	0.06	0.037	—	[0.01, 0.23]
Social media use time	0.20	0.05	< 0.001	—	[0.11, 0.30]
Physical neglect × social media use time	−0.07	0.06	0.237	0.711	[−0.20, 0.05]
Emotional abuse × social media use time	0.004	0.06	0.943	1.000	[−0.11, 0.12]
Emotional neglect × social media use time	−0.10	0.06	0.102	0.409	[−0.23, 0.02]
Physical abuse × social media use time	−0.02	0.06	0.745	1.000	[−0.14, 0.10]
Sexual abuse × social media use time	0.11	0.06	0.059	0.297	[−0.01, 0.23]

ASMC, Appearance-related social media consciousness; BMI, body mass index.

## 4 Discussion

This study, grounded in schema theory and objectification theory, examined the associations between different types of childhood maltreatment and ASMC, as well as the moderating roles of gender. Overall, the findings suggest that childhood maltreatment should not be treated as a homogeneous form of early adversity: its subtypes showed distinct associations with ASMC, including a negative conditional association for physical neglect; gender differences were confined to the sexual abuse–ASMC association, and no robust moderation by social media use time was detected. Specifically, in the overall model, emotional abuse and sexual abuse were positively associated with ASMC, whereas physical neglect was negatively associated with ASMC. Direct comparisons of the male and female coefficients indicated that only the association between sexual abuse and ASMC differed significantly by gender. In addition, social media use time was positively associated with ASMC, but did not significantly moderate the association between any childhood maltreatment subtype and ASMC after correction for multiple testing. Taken together, these findings partially support the theoretical expectations of the present study and highlight subtype-specific and gender-related patterns in the associations between childhood maltreatment and ASMC, while providing no evidence for a moderating role of social media use time.

### 4.1 Differential associations between childhood maltreatment subtypes and ASMC

The present findings provide support for Hypothesis 1, demonstrating that the associations between different subtypes of childhood maltreatment and ASMC are not uniform. Importantly, this pattern emerged not from simple bivariate associations, but from a model in which all five maltreatment subtypes were considered simultaneously, with their shared variance taken into account. Given the substantial co-occurrence among different forms of childhood maltreatment, examining any single subtype in isolation often makes it difficult to determine whether its association with ASMC reflects the specific effect of that subtype or the broader influence of accumulated childhood adversity (Herrenkohl and Herrenkohl, 2009). In contrast, the present results more clearly identify which maltreatment types retain unique explanatory power after the overlap among adverse childhood experiences has been controlled. Specifically, emotional abuse and sexual abuse remained significant positive predictors of ASMC, whereas physical neglect showed a significant negative association. These findings suggest that the effect of childhood maltreatment on ASMC is not a single cumulative-risk process, but a heterogeneous one in which different maltreatment subtypes may show distinct associations with ASMC.

Emotional abuse showed a unique positive association with ASMC, a pattern that is broadly consistent with schema theory. Schema theory posits that individuals gradually develop relatively stable cognitive structures about the self, others, and relationships through early interactions with significant others (Young et al., 2006). When children are repeatedly exposed to humiliation, rejection, devaluation, or denial, they may be more likely to develop maladaptive schemas characterized by feelings of inadequacy, expectations of criticism, and a stronger reliance on external validation. ASMC, however, reflects not merely concern with one’s appearance, but a continuing awareness of how one’s appearance may be evaluated by a social media audience (Choukas-Bradley et al., 2020), one possible interpretation is that emotional abuse may be associated with greater sensitivity to external evaluation, which could in turn be relevant to appearance-related concerns in highly visible social media contexts. This interpretation is also consistent with work showing that childhood maltreatment is linked to negatively bias self-referential processing and maladaptive schemas (Jopling et al., 2020). However, maladaptive schemas and evaluative sensitivity were not directly measured in the present study, and this account should therefore be regarded as a theoretical interpretation rather than a demonstrated mechanism.

The unique positive association between sexual abuse and ASMC is more consistent with objectification theory. According to objectification theory, repeated experiences of being viewed, scrutinized, or evaluated may lead individuals to internalize an observer’s perspective on their own bodies, thereby fostering self-objectification and habitual body surveillance (Fredrickson and Roberts, 1997). Prior research has also linked childhood maltreatment with later body-image difficulties (Bödicker et al., 2022). From this perspective, one possible interpretation is that experiences of sexual abuse may be associated with heightened attention to bodily and appearance-related evaluation, which may be relevant to ASMC in highly visual social media environments. However, the present study did not directly assess self-objectification, body surveillance, or the proposed processes linking sexual abuse to ASMC. Accordingly, objectification theory provides a possible interpretive framework for the observed association rather than evidence of a specific developmental pathway.

It is also noteworthy that physical neglect was negatively associated with ASMC only after adjustment for the other maltreatment subtypes. Physical neglect primarily reflects inadequate provision of basic physical care and support, and is conceptually distinct from emotional abuse and sexual abuse (Bernstein et al., 2003). In the sensitivity analysis, physical neglect was not significantly associated with ASMC when examined separately with BMI, whereas the negative association emerged in the mutually adjusted model. Although the variance inflation factors did not indicate serious multicollinearity, this pattern suggests that the negative coefficient for physical neglect is conditional on the shared variance among maltreatment subtypes and may represent a model-dependent or suppressor-like association. Accordingly, the present findings should not be interpreted as evidence that physical neglect is independently associated with lower ASMC. Rather, the negative coefficient should be viewed cautiously as a conditional association within the mutually adjusted model. Further research, particularly in samples with greater variability in physical neglect, is needed to determine the robustness and substantive meaning of this finding.

### 4.2 The moderating role of gender

The present findings partially supported Hypothesis 2. Although the omnibus multigroup comparison indicated that at least one of the five childhood maltreatment–ASMC paths differed by gender, the path-specific Wald tests showed that only the sexual abuse–ASMC association differed significantly between males and females. Among males, none of the five maltreatment subtypes was significantly associated with ASMC. Although the coefficient for emotional neglect was positive in direction, neither this within-group association nor the corresponding gender difference was statistically significant. Emotional neglect generally involves insufficient emotional attention, validation, and support during development, which may undermine a stable sense of self-worth and interpersonal security (Shorey et al., 2013). Moreover, traditional gender socialization may discourage males from expressing vulnerability or seeking emotional support (Addis and Mahalik, 2003; Berke et al., 2018). Although these perspectives provide a broader context for considering the relevance of emotional validation to appearance-related evaluation, the present findings do not support a male-specific compensatory pathway.

Among females, sexual abuse was positively associated with ASMC, whereas physical neglect was negatively associated with ASMC. Importantly, only the sexual abuse–ASMC association differed significantly between males and females. This gender difference may be cautiously interpreted in light of objectification theory, which proposes that girls and women are particularly likely to internalize an observer’s perspective on their bodies in sociocultural contexts characterized by appearance-based surveillance and evaluation (Fredrickson and Roberts, 1997). In the broader context of gendered appearance norms (Bödicker et al., 2022; Fredrickson and Roberts, 1997), experiences involving bodily violation may be associated with heightened vigilance concerning how one’s appearance is viewed and evaluated in highly visual social media environments. However, self-objectification, body surveillance, and related psychological processes were not directly assessed; therefore, this interpretation should be regarded as tentative rather than as a demonstrated mechanism.

Physical neglect primarily reflects failures to meet basic physical care and protection needs (Bernstein et al., 2003). Although it was negatively associated with ASMC within the female group, the corresponding male–female difference was not statistically significant. Moreover, physical neglect was not significantly associated with ASMC when examined separately in the sensitivity analysis, with the negative coefficient emerging only after adjustment for the other maltreatment subtypes. This pattern may reflect shared variance among maltreatment dimensions or a suppressor-like effect and should not be interpreted as evidence that physical neglect independently predicts lower ASMC or has a female-specific association with ASMC. Although different forms of childhood maltreatment may have distinct psychological correlates (Teicher and Samson, 2013), the present cross-sectional findings provide evidence of gender moderation only for the sexual abuse–ASMC association and do not establish the underlying developmental mechanisms.

### 4.3 The moderating role of social media use time

Social media use time was positively associated with ASMC; however, none of the interactions between social media use time and the five childhood maltreatment subtypes was statistically significant after correction for multiple testing. Thus, Hypothesis 3 was not supported. Previous research has shown that longer time spent on social media is generally associated with greater exposure to appearance-focused content, more frequent appearance comparisons, and more sustained engagement in image-based self-presentation and social interaction (Cohen et al., 2017; Cohen and Blaszczynski, 2015). The positive association observed in the present study is therefore broadly consistent with the view that greater engagement with social media may coincide with greater appearance-related consciousness. However, because the present measure assessed general social media use duration rather than appearance-focused exposure, this association should not be interpreted as evidence that longer use itself increases ASMC.

More importantly, the null interaction effects indicate that the associations between childhood maltreatment subtypes and ASMC did not systematically vary according to overall social media use time in the present sample. This finding is consistent with broader arguments that the psychological relevance of social media may depend not only on the amount of use, but also on the specific content encountered and the ways individuals engage with and process social media experiences (Fardouly and Vartanian, 2016; Kross et al., 2021; Orben and Przybylski, 2019). Future research may therefore benefit from using objective digital-trace measures and more content-specific indicators to examine whether particular forms of social media exposure moderate the associations between childhood maltreatment and ASMC.

### 4.4 Limitations

This study has several limitations. First, its cross-sectional design does not allow for strong causal inferences. Although childhood maltreatment temporally precedes current ASMC in principle, the present design cannot establish the developmental processes linking retrospectively reported childhood maltreatment to current ASMC. Future longitudinal research is therefore needed to examine these associations over time. Second, all variables were measured through self-report, which may introduce common method bias and retrospective recall bias, particularly in the assessment of childhood maltreatment. In addition, the physical abuse and sexual abuse subscales showed restricted distributions, with medians at the theoretical minimum and substantial positive skewness. Although robust estimation was used to address non-normality, it cannot compensate for the limited variability in these predictors. Findings involving these maltreatment subtypes should therefore be interpreted cautiously and replicated in samples with greater variability in maltreatment exposure. Third, social media use time was assessed with a single retrospective self-report item, which may be subject to reporting error and may not accurately correspond to objectively logged use. In addition, general social media use duration should not be interpreted as equivalent to exposure to appearance-focused content. Although this measure captures overall exposure, it cannot distinguish between different types of social media activities or usage patterns. Future research could therefore incorporate objective digital-trace measures and more fine-grained indicators, such as appearance-focused browsing, selfie posting, photo editing, and feedback seeking. Finally, because the sample consisted of Chinese college students, the generalizability of the findings to other populations remains to be established.

## 5 Conclusion

In conclusion, this study examined the associations between retrospectively reported childhood maltreatment and ASMC. In the mutually adjusted model, emotional abuse and sexual abuse were associated with higher levels of ASMC, whereas physical neglect showed a negative conditional association that emerged only after adjustment for the other maltreatment subtypes. Direct gender comparisons indicated that only the sexual abuse–ASMC association differed significantly between males and females. Social media use time was positively associated with ASMC but did not significantly moderate the associations between any of the five maltreatment subtypes and ASMC after correction for multiple testing. These cross-sectional findings highlight subtype-specific and gender-related patterns of association with ASMC but do not establish causal mechanisms or developmental pathways and require longitudinal replication.

## Data Availability

The raw data supporting the conclusions of this article will be made available by the authors, without undue reservation.
